# Aerobic exercise and DNA methylation in postmenopausal women: An ancillary analysis of the Alberta Physical Activity and Breast Cancer Prevention (ALPHA) Trial

**DOI:** 10.1371/journal.pone.0198641

**Published:** 2018-06-28

**Authors:** Devon J. Boyne, Will D. King, Darren R. Brenner, John B. McIntyre, Kerry S. Courneya, Christine M. Friedenreich

**Affiliations:** 1 Department of Cancer Epidemiology and Prevention Research, CancerControl Alberta, Alberta Health Services, Calgary, Alberta, Canada; 2 Department of Community Health Sciences, Cumming School of Medicine, University of Calgary, Calgary, Alberta, Canada; 3 Department of Public Health Sciences, Queen’s University, Kingston, Ontario, Canada; 4 Department of Oncology, Cumming School of Medicine, University of Calgary, Calgary, Alberta, Canada; 5 Translational Laboratory, Tom Baker Cancer Centre, Department of Pathology and Laboratory Medicine, University of Calgary, Calgary, Alberta, Canada; 6 Faculty of Physical Education and Recreation, University of Alberta, Edmonton, Alberta, Canada; Sapporo Ika Daigaku, JAPAN

## Abstract

Physical activity is associated with a lower risk of breast, colon, and endometrial cancer. Epigenetic mechanisms such as changes in DNA methylation may help to explain these protective effects. We assessed the impact of a one year aerobic exercise intervention on DNA methylation biomarkers believed to play a role in carcinogenesis. The Alberta Physical Activity and Breast Cancer Prevention (ALPHA) Trial was a two-armed randomized controlled trial in 320 healthy, inactive, postmenopausal women with no history of cancer. In an ancillary analysis, frozen blood samples (n = 256) were reassessed for levels of DNA methylation within LINE-1 and Alu repeats as well as within the promoter regions of *APC*, *BRCA1*, *RASSF1*, and *hTERT* genes. Differences between the exercise and control arm at 12-months, after adjusting for baseline values, were estimated within an intent-to-treat and per-protocol analysis using linear regression. No significant differences in DNA methylation between the exercise and control arms were observed. In an exploratory analysis, we found that the prospective change in estimated VO_2_max was negatively associated with *RASSF1* methylation in a dose-response manner (p-trend = 0.04). A year-long aerobic exercise intervention does not affect LINE-1, Alu, *APC*, *BRCA1*, *RASSF1*, or *hTERT* methylation in healthy, inactive, postmenopausal women. Changes in DNA methylation within these genomic regions may not mediate the association between physical activity and cancer in healthy postmenopausal women. Additional research is needed to validate our findings with *RASSF1* methylation.

**Trial Registration:** ClinicalTrials.gov NCT00522262.

## Introduction

Regular physical activity has been shown to protect against a multitude of cancers in a wide variety of study populations and settings. It is well established that being more physically active lowers the risk of colorectal, breast, and endometrial cancer [1, 2]. Emerging evidence suggests that exercise plays an important etiologic role in the prevention of other types of cancer as well. A recent pooled analysis of 1.44 million individuals suggested that the protective effects of physical activity also extend to head and neck, esophageal, lung, kidney, blood, and bladder cancers independent of body mass index [3]. The burden of cancer attributable to physical inactivity is considerable. It was recently estimated that roughly 7% of colorectal and breast cancer cases worldwide were attributable to physical inactivity in 2013, representing a total healthcare cost of $5.2 billion for these two cancer sites alone [4].

The underlying biologic mechanisms whereby physical activity influences cancer risk has been investigated in recent randomized controlled trials in healthy populations [5–9]. One recently identified but poorly understood mechanism whereby physical activity may prevent carcinogenesis is DNA methylation [10]. Patterns of DNA methylation can become dysregulated in response to ageing and exposure to carcinogenic agents which can lead to genomic instability and abnormal gene expression [11–13]. Such dysregulation is widely thought to be a molecular state that predisposes an individual to cancer [14, 15].

We invested the impact of a year-long exercise intervention on levels of DNA methylation within two major types of repetitive elements (LINE-1 and Alu) and within the promoter regions of four candidate genes (*APC*, *BRCA1*, *RASSF1*, and *hTERT*) in a group of healthy, inactive postmenopausal women. High levels of LINE-1 and Alu methylation are associated with chromosomal stability [16–18]. Although the evidence is conflicting and additional research is needed, some studies have found that individuals with lower levels of LINE-1 and Alu methylation in tissue or in blood have a higher risk of developing cancer [19–22]. We selected four genes based on evidence of an association between genetic mutations and epigenetic alterations in these genes and the risk of cancer. *APC*, *BRCA1*, and *RASSF1* are tumour suppressor genes which play important etiologic roles in carcinogenesis. High levels of methylation within the promoter regions of these genes is associated with gene expression silencing and is a common occurrence in several types of cancer tissue [23]. Recent epidemiologic studies have reported an increased risk of cancer among individuals with higher levels of *APC*, *BRCA1*, and *RASSF1* promoter methylation in tissue or in blood [24–28]. In contrast, elevated levels of methylation within the promoter region of *hTERT* is potentially associated with a protective carcinogenic effect via increased telomerase expression [29]. The *hTERT* gene encodes for the catalytic subunit of telomerase which regulates telomeric DNA length and plays a vital role in the cellular immortalization of cancers [30]. In healthy populations, shorter telomeres have been associated with an increased risk of cancer [31].

The objective of the current study was to determine if a year-long aerobic exercise intervention could impact blood-based measures of DNA methylation with LINE-1 and Alu regions as well as within the promoter regions of the *APC*, *BRCA1*, *RASSF1*, and *hTERT* genes. Although it has yet to be proven, we hypothesized that physical activity would have a systemic effect on levels of DNA methylation across all tissues such that any changes in DNA methylation detected within blood would also reflect changes in breast tissue–the target tissue of interest. We also hypothesized that higher levels of DNA methylation within LINE-1 and Alu regions and within the promoter region of the *hTERT* gene and that lower levels of DNA methylation within the promoter regions of the *APC*, *BRCA1*, and *RASSF1* genes would be associated with a reduced risk of cancer. As such, we hypothesized that women randomized to the exercise intervention would have significantly higher levels of LINE-1, Alu, and *hTERT* methylation and significantly lower levels of *APC*, *BRCA1*, and *RASSF1* methylation after adjusting for baseline differences.

## Methods

### Study population

Participant recruitment and eligibility have been detailed elsewhere [7]. To be eligible, participants had to be physically inactive postmenopausal women between the ages of 50 and 75 and non-smokers or excessive alcohol consumers. In addition, eligible participants had no prior history of cancer, had physician clearance to participate, and had to provide written informed consent. Ethics approval was obtained from the Alberta Cancer Research Ethics Committee, the University of Calgary, and the University of Alberta.

### Study design, intervention, and covariate information

The Alberta Physical Activity and Breast Cancer Prevention (ALPHA) Trial was a two-armed randomized year-long exercise intervention that assessed the effects of aerobic activity on biomarkers associated with breast cancer risk [7]. There were 320 participants who were randomized in a 1:1 ratio to either the intervention or control arm. The intervention began with 15–20 minutes of aerobic activity at 50–60% of the maximum heart rate three times/week. This exercise protocol was increased over the course of three months to 45 minutes of activity at 70–80% of the maximum heart rate five times/week which was sustained for the remaining nine months. A minimum of three weekly sessions were supervised at designated facilities while the remaining sessions were unsupervised.

Baseline and follow-up information on several covariates was collected. A baseline health questionnaire captured the participants’ age, ethnicity, and prior smoking history. Weight and height were objectively measured. Dietary folate intake, alcohol intake, and levels of physical activity in the year before study entry were estimated using validated questionnaires [32, 33]. At both baseline and follow-up, a submaximal exercise test was used to estimate maximal oxygen uptake (VO_2_max) as described previously [7].

### Blood collection and DNA methylation assays

Blood samples were collected from each participant at baseline (n = 320) and at 12-months (n = 310) after a ten hour fast using standardized collection, processing, and storage protocols [7]. In the Translational Laboratory at the Tom Baker Cancer Center (Calgary, Alberta), peripheral blood mononuclear DNA from 630 samples was purified and extracted using the Hamilton STARlet liquid handling instrument (Hamilton Robotics Inc., Reno, USA) and the Machery-Nagel NucleoMag Blood 200 μl kit (Macherey-Nagel GmbH & Co. KG, Düren, Germany). As determined by the Qubit dsDNA HS Assay Kit (ThermoFisher Scientific Inc., Waltham, USA), a total of 33 samples had insufficient DNA for further analysis. The remaining 597 samples were plated on seven 96-well plates. At the McGill University Génome Québec Innovation Centre (Montréal, Canada), the plated samples underwent sodium bisulfite conversion which was carried out with the EZ-96 DNA Methylation-Gold Kit (Zymo Research, Irvine, USA, Catalog No. D5007). DNA methylation within successfully treated samples (n = 573) was assessed using two methods. A pyrosequencing assay involving the HotStarTaq DNA Polymerase kit (Qiagen, Hilden, Germay) and PyroMark Q24 (Qiagen, Hilden, Germay) was used to measure the degree of LINE-1 and Alu methylation. The primers used in our assessment of LINE-1 and Alu methylation were developed in a prior investigation [34]. Methylation analyses for *APC*, *BRCA1*, *RASSF1* and *hTERT* were performed using Genome Quebec’s Sequenom® EpiTYPER platform and standard EpiPanel (Agena Bioscience, San Diego, USA). This mass spectrometry-based platform enables accurate and quantitative measurement of DNA methylation levels at multiple CpG genomic regions [35].The targeted regions and the corresponding CpG sites are described in S1 Table. Target Regions for Gene-Specific Outcomes.

### DNA methylation data processing

A total of 23, 43, 20, and 26 CpG sites were assessed within the promoter regions of the *APC*, *BRCA1*, *RASSF1*, and *hTERT* genes respectively. As done in previous studies [35, 36], we removed CpG sites deemed unreliable by any one of the following criteria: 1) the mass was beyond the limit of detection; 2) overlapping signals or silent peaks were detected; 3) there were multiple CpG units with the same mass; 4) the CpG unit had a low success rate which we defined as a failure rate of at least 10% across samples. After removing these unreliable CpG sites, there remained 7, 23, 13, and 21 CpG sites for the *APC*, *BRCA1*, *RASSF1*, and *hTERT* genes respectively (S1 Table. Spearman’s Correlation between Repetitive Element and Gene-Specific Baseline DNA Methylation Measures).

For each outcome, we excluded observations that were missing more than 15% of data within the remaining CpG sites. This step resulted in the exclusion of the following number of observations: LINE1 (n = 3), Alu (n = 3), *APC* (n = 28), *BRCA1* (n = 10), *RASSF1* (n = 3), and *hTERT* (n = 12). For both LINE1 and Alu methylation, we also excluded observations that failed to meet quality control standards specified by the PyroMark Q24 software (n = 3). Among the remaining observations, there were no missing methylation values for any of the CpG sites assessed within LINE1, Alu, or *APC*. Missingness within the other outcomes was addressed using a plate-specific mean imputation. For any given individual, no more than 3 CpG sites were imputed for the *hTERT* and *BRCA1* genes and no more than 1 CpG site was imputed for the *RASSF1* gene.

The overall level of DNA methylation within each genomic region of interest was defined as the average percent methylation across the remaining CpG sites. These raw values were adjusted for batch effects using the following mean centering approach: $y_{i}=(x_{i}-{\bar{x}}_{j})+\bar{y}$ where *y_i_* is the adjusted value for the ith observation, *x_i_* is the raw value for the ith observation, ${\bar{x}}_{j}$ is the mean specific to the jth batch, and $\bar{y}$ is the overall mean [37]. These batch adjusted methylation values were used in all subsequent analyses.

### Reliability assessment

We evaluated the inter-batch reliability for each DNA methylation measure. The samples were processed in seven batches. We had enough DNA to duplicate the laboratory analyses for eight samples. One of the seven batches was used as a reference batch. For each of the eight duplicated samples, we placed one of the samples in this reference batch. We then placed the paired duplicate samples in one of the other six remaining batches, in random order, such that each of the six remaining batches had at least one of the eight duplicate samples. Based on a visual examination of the Bland-Altman plots, one outlier was removed from the *BRCA1* and *APC* analyses. Inter-batch reliability was quantified using the coefficient of variation (CV) and limits of agreement (LOA) as follows: LINE1 (CV = 1.4%;LOA = -0.6–3.4), Alu (CV = 2.2%;LOA = -0.2–1.3), *APC* (CV = 8.6%;LOA = -0.1–0.6), *BRCA1* (CV = 9.8%;LOA = -0.2–0.7), *RASSF1* (CV = 15.0%;LOA = 0.0–1.3), and *hTERT* (CV = 10.8%;LOA = -2.3–8.1).

### Data analysis

The outcome of interest was the absolute mean difference in percent DNA methylation between the exercise arm and the control arm at follow-up after adjusting for baseline values. This effect was estimated using linear regression whereby follow-up methylation was modeled as a function of baseline methylation and treatment arm. *APC* methylation was log transformed to address non-normality. Intent-to-treat and per-protocol analyses were conducted. In the per-protocol analysis, individuals randomized to the intervention arm were excluded if they did not adhere to at least 90% of the target exercise time. Within the intent-to-treat framework, subgroup analyses were conducted by testing the significance of the interaction term between treatment arm and the following covariates: age (<60/≥60 years), BMI (normal:18.5–24.9 kg/m^2^; overweight:25.0–29.9 kg/m^2^; obese:30.0+ kg/m^2^), baseline VO_2_max (above/below the median), and family history of breast cancer (yes/no).

As an exploratory analysis, we also investigated the association between the amount of physical activity, weight loss, and change in aerobic fitness that occurred during the intervention and differences in DNA methylation at 12-months.

Individuals with incomplete DNA methylation data were not included in the foregoing analyses. We assumed that these data were missing completely at random since any missingness would be due to shortcomings in laboratory methods. To assess the robustness of our results, we conducted a sensitivity analysis whereby the analyses were repeated after imputing DNA methylation values among individuals with partial outcome information. A multiple imputation procedure was used to impute ten datasets using DNA methylation and baseline covariate data as auxiliary variables. SAS v.9.4 was used for all analyses.

## Results

**Fig 1:**
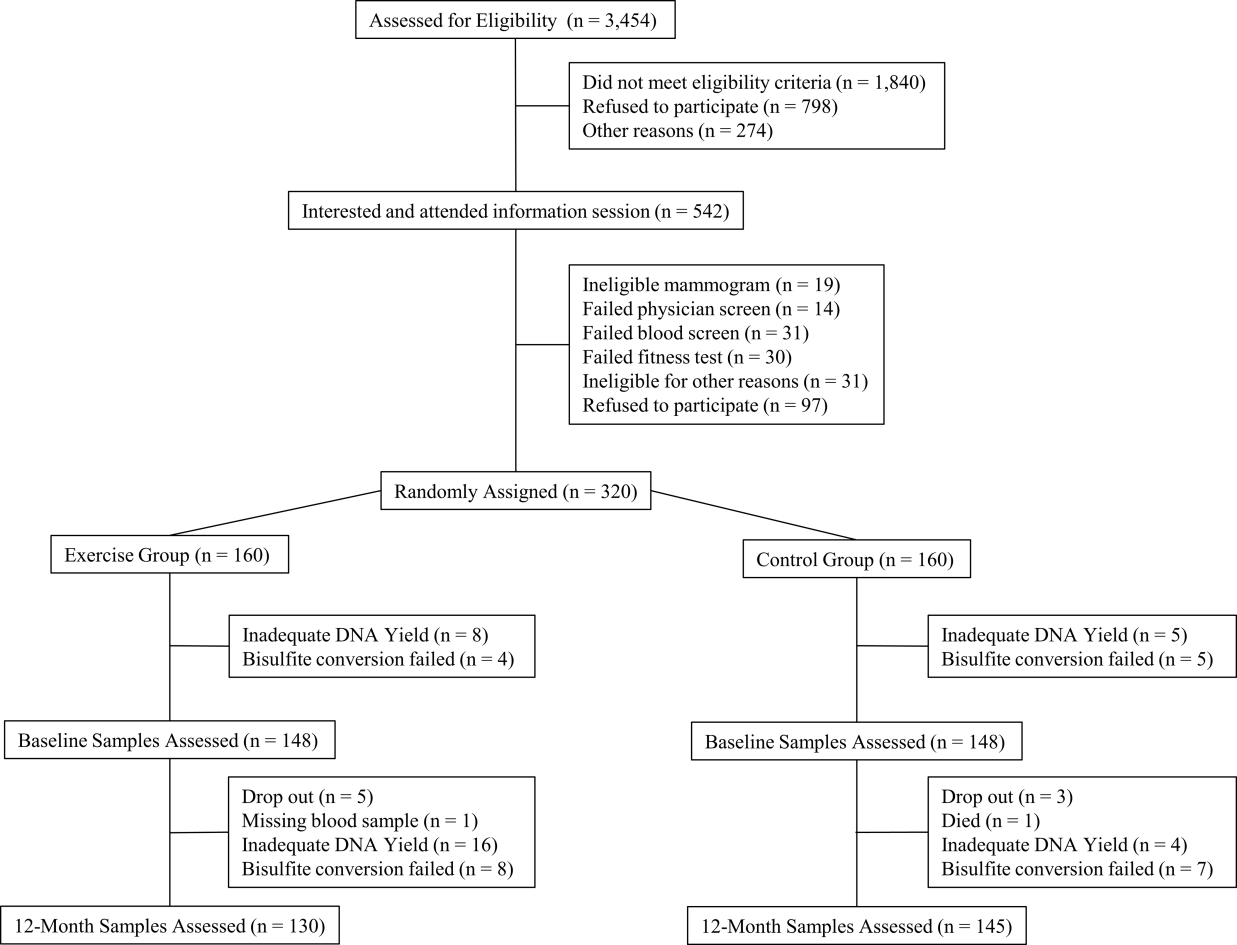
CONSORT diagram of ALPHA trial ancillary study. This Consolidated Standards of Reporting Trials (CONSORT) diagram describes the inclusion and exclusion of participants included in the current trial.

The flow of participants through the study and the numbers available for analysis are summarized in Fig 1. The two randomized groups were balanced with respect to baseline covariates (Table 1). The women included in these analyses had a mean age of 61.1 years (range = 50.6–74.9), a BMI of 29.2 kg/m^2^ (range = 20.4–43.9), and VO_2_max of 26.7 ml/kg/min (range = 11.5–52.2). Most of these participants (91.8%) were Caucasian.

**Table 1:** Baseline characteristics of ALPHA trial participants included in intent-to-treat analysis.

Baseline Characteristic	Exercise (N = 122)	Control (N = 134)
Age (years), mean (SD)	61.5 (5.4)	60.7 (5.6)
Caucasian, n (%)	109 (90.1)^a^	125 (93.3)
Never smoker, n (%)	88 (75.9)^b^	82 (63.1)^c^
Body mass index (kg / m^2^), mean (SD)	28.8 (4.2)	29.6 (4.3)
Weight (kg), mean (SD)	74.7 (12.3)	77.4 (12.9)
Total activity (MET-hours/week), mean (SD)	116.7 (60.7)	134.4 (81.9)
Physical fitness (VO_2max_, ml/kg/min), mean (SD)	26.8 (6.2)	26.6 (6.1)
Alcohol (g/day), median (IQR)	4.6 (6.0)^d^	5.1 (7.9)^a^
Folate (mcg DFE/day), median (IQR)^e^	405.7 (175.7)^d^	425.6 (191.2)^a^

^a^ N miss = 1

^b^ N miss = 6

^c^ N miss = 4

^d^ N miss = 3

^e^ Dietary folate equivalents (DFE)

A weak positive correlation was found between baseline LINE1 and Alu methylation (Spearman’s rho = 0.15, p<0.01). We did not detect a statistically significant correlation between any of the other DNA methylation measures at baseline (S2 Table).

The results of the intent-to-treat and per-protocol analyses are summarized in Tables 2 and 3 respectively. In both analyses, we did not detect a statistically significant difference in DNA methylation between the exercise and the control arms at follow-up after adjusting for baseline methylation values. The magnitude of the observed effect in these analyses was small–the absolute mean difference in estimated percent methylation between the two groups was less than 0.5% for all outcomes.

**Table 2:** Intent-to-treat analysis comparing changes in the DNA methylation outcomes between the exercise and control groups.

	N	Baseline^a^	12-Months^a^	Difference at 12-Months^b^	P-value
**LINE1**					
Control	131	72.85 (72.59 to 73.10)	73.01 (72.72 to 73.30)	Ref.	
Exercise	120	72.83 (72.54 to 73.12)	72.78 (72.54 to 73.02)	-0.23 (-0.60 to 0.14)	0.23
**Alu**					
Control	131	19.11 (19.03 to 19.19)	19.09 (19.00 to 19.17)	Ref.	
Exercise	120	19.09 (19.01 to 19.18)	19.12 (19.05 to 19.19)	+0.03 (-0.08 to 0.14)	0.57
**APC**^c^					
Control	124	1.76 (1.65 to 1.88)	1.73 (1.61 to 1.85)	Ref.	
Exercise	109	1.79 (1.68 to 1.91)	1.68 (1.58 to 1.78)	0.97 (0.88 to 1.06)^c^	0.52
**BRCA1**^d^					
Control	128	1.76 (1.68 to 1.85)	1.79 (1.71 to 1.87)	Ref.	
Exercise	117	1.77 (1.69 to 1.84)	1.74 (1.66 to 1.81)	-0.02 (-0.09 to 0.05)	0.60
**RASSF1**					
Control	133	3.33 (3.24 to 3.42)	3.22 (3.14 to 3.31)	Ref.	
Exercise	121	3.26 (3.17 to 3.35)	3.21 (3.11 to 3.30)	-0.01 (-0.14 to 0.11)	0.84
**hTERT**					
Control	132	17.32 (16.73 to 17.90)	17.58 (16.96 to 18.20)	Ref.	
Exercise	117	16.64 (16.08 to 17.21)	17.55 (16.88 to 18.23)	+0.20 (-0.67 to 1.07)	0.65

^a^ Estimated mean (95% C.I.) percent methylation

^b^ Estimated mean difference (95% C.I.) in percent methylation between the exercise arm and control arm at follow-up after adjusting for baseline methylation

^c^ The data were log transformed to address non-normality. Presented are the geometric means (95% C.I.) at baseline and 12-months. The estimated difference is the ratio of the geometric means of the exercise arm and control arm at follow-up after adjusting for baseline methylation.

^d^ Four influential observations excluded from analysis (n exercise = 2; n control = 2)

**Table 3:** Per-protocol analysis comparing changes in the DNA methylation outcomes between the exercise and control groups.

	N	Baseline^a^	12-Months^a^	Difference at 12-Months^b^	P-value
**LINE1**					
Control	131	72.85 (72.59 to 73.10)	73.01 (72.72 to 73.30)	Ref.	
Exercise^c^	79	72.86 (72.48 to 73.23)	72.83 (72.52 to 73.14)	-0.18 (-0.62 to 0.26)	0.42
**Alu**					
Control	131	19.11 (19.03 to 19.19)	19.09 (19.00 to 19.17)	Ref.	
Exercise^c^	79	19.13 (19.02 to 19.23)	19.13 (19.03 to 19.23)	+0.04 (-0.09 to 0.18)	0.51
**APC**^d^					
Control	124	1.76 (1.65 to 1.88)	1.73 (1.61 to 1.85)	Ref.	
Exercise^c^	72	1.80 (1.66 to 1.96)	1.68 (1.56 to 1.81)	0.97 (0.87 to 1.08)^d^	0.59
**BRCA1**^e^					
Control	128	1.76 (1.68 to 1.85)	1.79 (1.71 to 1.87)	Ref.	
Exercise^c^	76	1.69 (1.60 to 1.77)	1.75 (1.67 to 1.83)	+0.04 (-0.04 to 0.12)	0.36
**RASSF1**					
Control	133	3.33 (3.24 to 3.42)	3.22 (3.14 to 3.31)	Ref.	
Exercise^c^	79	3.28 (3.17 to 3.39)	3.18 (3.07 to 3.29)	-0.04 (-0.18 to 0.09)	0.55
**hTERT**					
Control	132	17.32 (16.73 to 17.90)	17.58 (16.96 to 18.20)	Ref.	
Exercise^c^	77	16.79 (16.06 to 17.52)	17.67 (16.78 to 18.57)	+0.29 (-0.71 to 1.29)	0.57

^a^ Estimated mean (95% C.I.) percent methylation

^b^ Estimated mean difference (95% C.I.) in percent methylation between the exercise arm and control arm at follow-up after adjusting for baseline methylation

^c^ Excluded participants who did not adhere to 90% of target exercise time (i.e. an average of 180 min/week of moderate to vigorous aerobic activity over 12-months)

^d^ The data were log transformed to address non-normality. Presented are the geometric means (95% C.I.) at baseline and 12-months. The estimated difference is the ratio of the geometric means of the exercise arm and control arm at follow-up and after adjusting for baseline methylation.

^e^ Four influential observations excluded from analysis (n exercise = 2; n control = 2)

In subgroup analyses, no significant modification of the intervention effect by baseline age, BMI, estimated VO_2_max, or family history of breast cancer was observed for any outcome (p-interaction>0.05;data not shown).

The exploratory analyses examining the association between estimated change in VO_2_max and DNA methylation (Table 4) revealed a statistically significant negative dose-response association between change in physical fitness and *RASSF1* methylation at 12-months after adjusting for age and baseline values (p-trend = 0.04). Weight loss and time spent exercising were not associated with any of the DNA methylation outcomes (Table 4).

**Table 4:** The association between the amount of exercise, weight loss, change in VO_2_ max and DNA methylation during a year-long aerobic physical activity intervention.

	Estimated Mean Difference at 12-months Adjusted for Age and Baseline Values (95% CI)											
	N	LINE-1	N	Alu	N	APC^a^	N	BRCA1^b^	N	RASSF1	N	hTERT
**Time Exercising**^c^												
Controls	131	Ref.	131	Ref.	124	Ref.	129	Ref.	133	Ref.	132	Ref.
<150 min/wk	30	-0.41 (-1.01 to 0.19) p = 0.18	30	0.05 (-0.13 to 0.23) p = 0.61	25	0.90 (0.77 to 1.05) p = 0.16	29	-0.15 (-0.27 to -0.02) p = 0.02	30	0.09 (-0.11 to 0.30) p = 0.36	29	0.14 (-1.27 to 1.55) p = 0.84
≥150 to <225 min/wk	50	-0.07 (-0.56 to 0.43) p = 0.79	50	-0.02 (-0.17 to 0.13) p = 0.80	49	0.95 (0.85 to 1.07) p = 0.43	50	-0.01 (-0.12 to 0.09) p = 0.81	51	-0.10 (-0.26 to 0.07) p = 0.26	49	-0.02 (-1.18 to 1.13) p = 0.97
≥225 min/wk	40	-0.27 (-0.81 to 0.26) p = 0.36	40	0.09 (-0.07 to 0.25) p = 0.29	35	1.04 (0.90 to 1.19) p = 0.60	39	0.08 (-0.04 to 0.19) 0.18	40	0.02 (-0.17 to 0.20) p = 0.87	39	0.39 (-0.86 to 1.64) p = 0.54
*p-trend*^d^		0.33		0.48		0.99		0.38		0.70		0.64
**Weight Loss**^e^												
Gain (>3%)	26	Ref.	26	Ref.	25	Ref.	25	Ref.	26	Ref.	26	Ref.
Maintenance (+/-3%)	142	-0.09 (-0.73 to 0.54) p = 0.78	142	-0.05 (-0.24 to 0.14) p = 0.63	132	1.08 (0.92 to 1.25) p = 0.35	140	0.04 (-0.09 to 0.17) p = 0.57	141	0.01 (-0.21 to 0.22) p = 0.96	142	-0.72 (-2.18 to 0.73) p = 0.33
Minor loss(>3% to <5%)	27	-0.17 (-0.99 to 0.65) p = 0.69	27	-0.02 (-0.27 to 0.23) p = 0.87	26	1.03 (0.85 to 1.25) p = 0.77	27	0.03 (-0.14 to 0.19) p = 0.75	27	0.04 (-0.24 to 0.31) p = 0.80	27	-0.03 (-1.90 to 1.85) p = 0.98
Meaningful loss (≥5%)	54	0.00 (-0.71 to 0.71) p = 0.99	54	-0.10 (-0.31 to 0.11) p = 0.36	47	1.09 (0.92 to 1.29) p = 0.34	54	-0.02 (-0.17 to 0.12) p = 0.75	55	0.02 (-0.22 to 0.26) p = 0.86	53	0.21 (-1.42 to 1.85) 0.83
*p-trend*^f^		0.64		0.90		0.75		0.68		0.80		0.95
**Physical Fitness**^g^												
Meaningful decrease (≥10%)	40	Ref.	40	Ref.	41	Ref.	40	Ref.	42	Ref.	41	Ref.
Moderate to no change (+/-9.9%)	100	+0.02 (-0.55 to 0.58) p = 0.95	100	+0.06 (-0.11 to 0.23) p = 0.47	92	0.90 (0.79 to 1.02) p = 0.10	99	-0.13 (-0.24 to -0.02) p = 0.02	99	-0.06 (-0.24 to 0.13) p = 0.56	99	+0.85 (-0.44 to 2.14) p = 0.20
Meaningful increase (≥10%)	101	+0.32 (-0.24 to 0.88) p = 0.26	101	+0.07 (-0.10 to 0.23) p = 0.44	90	0.93 (0.81 to 1.05) p = 0.24	98	-0.10 (-0.21 to 0.01) p = 0.09	102	-0.17 (-0.35 to 0.02); p = 0.07	100	+0.39 (-0.89 to 1.67) p = 0.65
*p-trend*^h^		0.15		0.53		0.46		0.29		0.04		0.90

^a^ APC methylation was log-transformed to address non-normality. Presented are the estimated ratio (95% CI) of the geometric means at 12-months after adjusting for baseline differences

^b^ Two influential observations were excluded from the analysis

^c^ The average amount of moderate to vigorous aerobic activity completed during the intervention

^d^ Assessed by modeling the rank of the categories as a measured variable

^e^ The amount of weight lost during the intervention as a percentage of the participant’s baseline weight

^f^ Assessed by modeling the median cut-points (gain = 4.51%; maintenance = 0.16%; minor loss = 4.23%; meaningful loss = 7.31%) as a measured variable

^g^ The change in VO_2_ max that occurred during the intervention as a percentage of the participant’s baseline VO_2_ max

^h^ Assessed by modeling the median cut-points (decrease = -16.80%; no = +1.62%; increase = 24.34%) as a measured variable

After imputing values for individuals with incomplete DNA methylation data, similar results for the intent-to-treat and per-protocol analyses were obtained (data not shown). In the exploratory analysis, however, the dose-response association between change in VO_2_max and *RASSF1* methylation was attenuated and no longer statistically significant (p-trend = 0.16). Compared to those who had a meaningful decrease in VO_2_max, the estimated mean difference at 12-months in *RASSF1* percent methylation was -0.04 (95% CI:-0.23–0.14) and -0.12 (95% CI:-0.31–0.07) for individuals who had moderate to no change and for those who had a meaningful increase respectively. No other noteworthy differences between the complete-case and multiple imputation analyses were observed.

## Discussion

In this group of healthy, inactive, postmenopausal women, a year-long exercise intervention did not have a significant impact on levels of DNA methylation within LINE-1 and Alu repeats or within the promoter regions of *APC*, *BRCA1*, *RASSF1*, or *hTERT*. The current state of epidemiologic evidence pertaining to the epigenetic effects of physical activity is disparate and has been previously reviewed [10, 38–40]. Few candidate gene studies targeting *APC*, *BRCA1*, *RASSF1*, or *hTERT* have been conducted [40]. Our null findings with respect to *APC*, *BRCA1*, and *RASSF1* are consistent with results from two previous cross-sectional candidate gene studies in healthy women [41, 42] and several epigenome-wide association studies [40]. These null results might suggest that DNA methylation within these genes is tightly regulated in healthy individuals and is resistant to environmental influence.

In contrast to our gene-specific measures, a greater number of investigations on the epigenetic effects of physical activity have focused on repetitive element DNA methylation, particularly LINE-1 methylation [40]. Consistent with our findings, a year-long randomized weight loss trial found that a dietary and exercise intervention did not impact LINE-1 methylation in a group of overweight sedentary women [43]. Similar null findings have also been reported in various observational settings [44, 45]. However, White et al. conducted a large cross-sectional study on 647 middle aged Caucasian women with a family history of breast cancer and found that childhood, teenage, and adulthood levels of physical activity were positively associated with LINE-1 methylation [46]. One possible explanation for this disparity may be the duration and timing of the exposure. The studies reporting null findings have assessed levels of physical activity over relatively short periods of time, ranging from four-day accelerometer assessments to one-year exercise interventions or past-year levels of physical activity. In contrast, White et. Al (2013) assessed lifetime levels of physical activity. These findings may suggest that LINE-1 methylation is relatively impervious to short term changes in physical activity and is more largely determined by levels of physical activity sustained over long periods of time.

In an exploratory analysis, we found a significant positive dose-response association between changes in VO_2_max and *RASSF1* methylation. The direction of this finding is consistent with a cancer prevention mechanism. However, the magnitude of the estimated mean difference in *RASSF1* methylation at 12-months between the highest and lowest categories of change in VO_2_max was 0.17% which may not be clinically meaningful. In addition, this analysis was post-hoc, the dose-response association was no longer statistically significant after imputing DNA methylation values for individuals with incomplete data, and multiple comparisons were made. As such, the finding of an association between VO_2_max and *RASSF1* methylation should strictly be considered hypothesis-generating.

The weaknesses of this study should be acknowledged. The current study was an ancillary analysis of the ALPHA trial which was not originally designed to explore changes in DNA methylation. As such, we were forced to rely on the use of blood samples in our analyses which is a major limitation of this study. Specifically, the lack of an association between exercise and DNA methylation that we observed within blood may not reflect a lack of an association within breast, colon, endometrial, and other tissues of greater etiologic interest [47]. We were unable to estimate the distribution of white blood cell types in our analysis and measurement error arising from failure to adjust for cell-type distribution could partially account for our null findings [48]. Finally, the exclusion criteria used in the current study limits the generalizability of these results.

The strengths of this investigation should also be noted. The risk of bias due to confounding has been minimized through randomization. There was minimal attrition in this study since only nine (2.8%) participants were lost to follow-up for reasons unrelated to the intervention. Lastly, the intervention was also highly successful with respect to increasing physical activity levels. Among the individuals included in our intent-to-treat analysis, 70.5% completed 150+ min/week of exercise over the course of the intervention.

Future research should consider assessing levels of physical activity over extended periods of time. Studies may also wish to target more heterogeneous study populations to allow for the exploration of modification by cancer risk profile. DNA methylation investigations should ideally be carried out within the target tissue of interest and those conducted in blood should estimate the distribution of blood cells directly or should consider indirect estimation methods when available [49]. In addition, future studies could explore the association between physical activity and DNA methylation within genomic regions other than those investigated in the current analysis. Rather than focusing on cancer prevention, researchers could also consider exploring the mediating role of DNA methylation with respect to physical activity and cancer survival as previously done. [50]

## Conclusion

A year-long aerobic exercise intervention did not impact levels of DNA methylation within LINE-1 and Alu repeats or within the promotor regions of the *APC*, *BRCA1*, *RASSF1*, and *hTERT* genes in the white blood cells of healthy, inactive, postmenopausal women. An exploratory analysis suggested that changes in physical fitness may be negatively associated with changes in *RASSF1*. Future large-scale studies examining changes in physical activity over longer periods of time in diverse study populations are required to validate these findings.

## Supporting information

S1 Table Target Regions for Gene-Specific Outcomes. (DOCX)

S2 Table Spearman’s correlation between Repetitive Element and Gene-Specific Baseline DNA Methylation Measures. (DOCX)
