# Supplementary material for: Aerobic exercise and DNA methylation in postmenopausal women: An ancillary analysis of the Alberta Physical Activity and Breast Cancer Prevention (ALPHA) Trial
Source: PLoS One. 2018 Jun 28;13(6):e0198641. doi: 10.1371/journal.pone.0198641 (PMC6023230; doi:10.1371/journal.pone.0198641)
Supplement: S1 Table — (DOCX) [file pone.0198641.s001.docx]

**S1 Table. Target Regions for Gene-Specific Outcomes**

| **Gene** | **GenBank**  **Accession** | **Fragment** | **Target Region** | **Total CpG Sites^a^ (#)** | **CpG Sites Included (#)^b^** | **Position^c^** |
| --- | --- | --- | --- | --- | --- | --- |
| *APC* | NG_008481.4 | 1 | chr5:112073349 to 112073614 | 23 | 7 | 112073365, 112073427, 112073434, 112073439, 112073535, 112073571, 112073614 |
|  |  |  |  |  |  |  |
| *BRCA1* | NG_005905.2 | 1 | chr17:41278054 to 41277665 | 15 | 5 | 41277940, 41277925, 41277868, 41277762, 41277694 |
|  |  | 2 | chr17:41277596 to 41277364 | 17 | 12 | 41277573, 41277541, 41277487, 41277444, 41277428, 41277426, 41277400, 41277394, 41277392, 41277389, 41277381, 41277364 |
|  |  | 3 | chr17:41277322 to 41277105 | 11 | 8 | 41277322, 41277231, 41277213, 41277186, 41277152, 41277116, 41277114, 41277105 |
|  |  |  |  |  |  |  |
| *hTERT* | NG_009265.1 | 1 | chr5:1295771 to 1295586 | 26 | 21 | 1295761, 1295759, 1295753, 1295737, 1295731, 1295725, 1295715, 1295713, 1295707, 1295705, 1295699, 1295674, 1295665, 1295655, 1295650, 1295648, 1295644, 1295618, 1295605, 1295593, 1295590 |
|  |  |  |  |  |  |  |
| *RASSF1* | NG_023270.1 | 1 | chr3:50378347 to 50378540 | 20 | 13 | 50378347, 50378350, 50378371, 50378373, 50378408, 50378414, 50378424, 50378426, 50378432, 50378493, 50378497, 50378516, 50378540 |

^a^ Total number of CpG sites assessed

^b^ Number of CpG sites included in analysis

^c^ Position of CpG sites included in analysis
