# Supplementary material for: Aerobic exercise and DNA methylation in postmenopausal women: An ancillary analysis of the Alberta Physical Activity and Breast Cancer Prevention (ALPHA) Trial
Source: PLoS One. 2018 Jun 28;13(6):e0198641. doi: 10.1371/journal.pone.0198641 (PMC6023230; doi:10.1371/journal.pone.0198641)
Supplement: S2 Table — (DOCX) [file pone.0198641.s002.docx]

**S2 Table. Spearman’s Correlation between Repetitive Element and Gene-Specific Baseline DNA Methylation Measures**

|  | **LINE-1** | **Alu** | **APC** | **BRCA1** | **RASSF1** | **hTERT** |
| --- | --- | --- | --- | --- | --- | --- |
| **LINE-1** |  | r = 0.15  p = 0.01  n = 293 | r = 0.05  p = 0.36  n = 284 | r = -0.03  p = 0.64  n = 290 | r = 0.08  p = 0.19  n = 292 | r = 0.10  p = 0.10  n = 292 |
| **Alu** | r = 0.15  p = 0.01  n = 293 |  | r = -0.11  p = 0.06  n = 284 | r = -0.11  p = 0.06  n = 290 | r = 0.06  p = 0.31  n = 292 | r = -0.03  p = 0.61  n = 292 |
| **APC** | r = 0.05  p = 0.36  n = 284 | r = -0.11  p = 0.06  n = 284 |  | r = 0.08  p = 0.20  n = 283 | r = -0.09  p = 0.11  n = 287 | r = 0.09  p = 0.12  n = 284 |
| **BRCA1** | r = -0.03  p = 0.64  n = 290 | r = -0.11  p = 0.06  n = 290 | r = 0.08  p = 0.20  n = 283 |  | r = 0.02  p = 0.78  n = 291 | r = 0.04  p = 0.49  n = 289 |
| **RASSF1** | r = 0.08  p = 0.19  n = 292 | r = 0.06  p = 0.31  n = 292 | r = -0.09  p = 0.11  n = 287 | r = 0.02  p = 0.78  n = 291 |  | r = 0.09  p = 0.11  n = 292 |
| **hTERT** | r = 0.10  p = 0.10  n = 292 | r = -0.03  p = 0.61  n = 292 | r = 0.09  p = 0.12  n = 284 | r = 0.04  p = 0.49  n = 289 | r = 0.09  p = 0.11  n = 292 |  |
